# Synthesis and Chromatography-Free Purification of PNA-PEO Conjugates for the Functionalisation of Gold Sensors

**DOI:** 10.3390/molecules170911026

**Published:** 2012-09-13

**Authors:** Monica Dettin, Davide Silvestri, Roberta Danesin, Erica Cretaio, Gianluca Picariello, Elisabetta Casarin, Agnese Sonato, Filippo Romanato, Margherita Morpurgo

**Affiliations:** 1 Department of Industrial Engineering, University of Padova, via Marzolo, 9, 35131 Padova, Italy; Email: monica.dettin@unipd.it (M.D.); roberta.danesin@unipd.it (R.D.); 2 Department of Pharmaceutical Chemistry and Pharmacology, University of Padova, via Marzolo, 5, 35131 Padova, Italy; Email: davide.silvestri@studenti.unipd.it (D.S.); elisabetta.casarin.1@unipd.it (E.C.); 3 Inter-University Consortium of Veneto for Nanotechnology (CIVEN), via delle Industrie, 5, 30174 Venezia, Italy; Email: cretaio@civen.org; 4 Institute of Food Sciences, CNR, via Roma, 64, 83100 Avellino, Italy; Email: picariello@isa.cnr.it; 5 Physics Department, University of Padova, via Marzolo, 8, 35131 Padova, Italy; Email: agnese.sonato@studenti.unipd.it (A.S.); filippo.romanato@unipd.it (F.R.)

**Keywords:** PEG, PEO, PNA, PNA-conjugate, surface plasmon resonance

## Abstract

Peptide Nucleic Acids (PNAs) linked to high molecular weight (MW) poly(ethylene oxide) (PEO) derivatives could be useful conjugates for the direct functionalisation of gold surfaces dedicated to Surface Plasmon Resonance (SPR)-based DNA sensing. However their use is hampered by the difficulty to obtain them through a convenient and economical route. In this work we compared three synthetic strategies to obtain PNA-high MW PEO conjugates composed of (a) a 15-mer PNA sequence as the probe complementary to genomic DNA of *Mycobacterium tuberculosis*, (b) a PEO moiety (2 or 5 KDa MW) and (c) a terminal trityl-protected thiol necessary (after acidic deprotection) for grafting to gold surfaces. The 15-mer PNA was obtained by solid-phase synthesis. Its amino terminal group was later condensed to bi-functional PEO derivatives (2 and 5 KDa MW) carrying a Trt-cysteine at one end and a carboxyl group at the other end. The reaction was carried out either in solution, using HATU or PyOxim as coupling agents, or through the solid-phase approach, with 49.6%, 100% and 5.2% yield, respectively. A differential solvent extraction strategy for product purification without the need for chromatography is described. The ability of the 5 KDa PEO conjugate to function as a probe for complementary DNA detection was demonstrated using a Grating-Coupling Surface Plasmon Resonance (GC-SPR) system. The optimized PEO conjugation and purification protocols are economical and simple enough to be reproduced also within laboratories that are not highly equipped for chemical synthesis.

## 1. Introduction

The efficacy of any surface-based biosensor strongly depends on the quality of its surface chemistry [1], which must ensure high affinity for the target analyte and prevent non-specific adsorption of compounds that are co-present in the biological sample. The issue of non-specific adsorption is particularly relevant in the case of label-free detection methods, as for example, those that rely on Surface Plasmon Resonance (SPR). Surface Plasmons (SP) are electron waves generated at the interface between a metal (most commonly gold) and a dielectric medium, upon interaction with light. The resonance of SPs, being highly sensitive to the light incident angle, is affected by the refractive index of the surface-localized dielectric medium. Any biorecognition event occurring in proximity of the surface induces a change in the local dielectric medium and therefore, if the event occurs on a SP generating surface, it can be transduced into a quantitative signal. This occurs without the need for further labeling of the surface-captured analyte, as otherwise necessary in other biosensing technologies (e.g., those relying on fluorescence or enzyme-linked color or chemiluminescent reactions). This represents an important advantage but also a potential pitfall since the signal generated is associated to the amount of material that becomes associated to the surface, independently of its nature. As a consequence, if non-specific adsorptions are not efficiently shielded, their contribution to the signal cannot be distinguished from that of the analyte. Therefore, the quality of a SPR sensing surface strongly depends on its ability to resist to non-specific adsorptions. In addition, as in any surface based sensor, two other elements are also fundamental: (a) the sensitivity and specificity of the sensing probes tethered to the surface; (b) the chemistry for surface bonding, that should favor the probe optimal orientation leaving its biorecognition ability unchanged.

With respect to the prevention of non-specific binding, it is widely accepted that a hydrophilic layer must be inserted between the probe and the sensing surface. Several hydrophilic polymers are used, among which cellulose [2], carboxymethyldextran [3,4], acrylamide [5] or poly(ethylene oxide) (PEO) [6,7]. However, most polymers require the use of multi-step procedures to build the whole sensing layer, in which the non fouling moiety is added first, followed by the probe which can be linked to the first layer through different chemical approaches [6,8,9,10,11,12,13,14,15]. Unfortunately, multi-step procedures share the common drawback of being tedious and not always reproducible, due to the intrinsic variability in efficiency of most coupling reactions which are strongly affected by the local environment (temperature, humidity, pH, local concentration of reagents) [16]. A single step functionalisation strategy is therefore advisable [17], in which the coupling efficacy is not highly influenced by the environment, thus allowing good reproducibility among functionalised surface replicas. This is achievable by using PEO derivatives in which one end is transformed to become reactive for surface tethering, the other one is linked to the biorecognizing moiety. 

In the case of DNA sensing, it is possible to improve the efficacy of the DNA capturing probe by using DNA analogues with superior properties than DNA itself. One example are the Peptide Nucleic Acids (PNAs) [18,19,20], pseudopeptide nucleic acid structural mimics, consisting of a backbone of repeating *N-*(2-aminoethyl)glycine units linked by peptide bonds and bearing nucleotide bases. When compared to their DNA or RNA analogues, PNAs possess higher affinity for sequence-complementary oligonucleotides, are chemically more stable and allow better discrimination between fully complementary and single-base mismatched DNA sequences [11,21,22,23,24].

Custom synthesis of PEO conjugated PNA probes suitable for surface anchorage is indeed offered by peptide and PNA manufacturers. However, the PEO derivatives inserted by these manufacturers are low molecular weight (MW) ones, while PEO polymer lengths ≥ 2 KDa are considered the most efficient ones in preventing non-specific binding [25,26]. PNA conjugates with low molecular weight PEO are relatively easy to insert by the solid phase approach, whereas, larger MW PEOs conjugates are hard to obtain by the same procedure due to steric hindrances. A high MW PEO-PNA conjugate synthesis was described in the literature [27] but very few technical details on the synthesis and purification and no information on reaction yields were given, so that obtaining this sort of compounds remains challenging for most laboratories. As a consequence, high MW-PEO-PNA surface tethering has so far been obtained through multi-step approaches only [6].

**Scheme 1:**
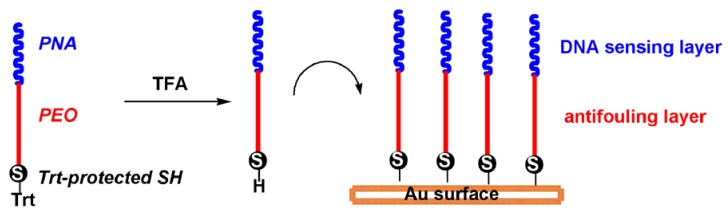
The designed PNA-PEO conjugate and the strategy for its binding to gold.

In this work we compare two solution and one solid-phase approaches to condense 15mer PNA with high MW PEOs (2 and 5 KDa) derivatives in order to produce conjugates for one-step functionalisation of gold surfaces dedicated to SPR DNA sensing. An amino-terminal free 15-mer PNA was initially synthesized via the solid-phase approach and was later condensed via peptide bond formation with an end-terminal PEO carboxyl group. The second end of the PEO was preventively functionalised with Fmoc-Cys(Trt)-OH to allow, after Trt deprotection, conjugation of the PNA-PEO molecule to a gold chip surface (Scheme 1).

The procedures here used for solution bioconjugation have been selected so to be economical and to be reproducible also within laboratories that are not highly equipped to perform chemical synthesis. The 5 KDa PEO conjugate prepared according to this procedure was used as a probe for complementary DNA detection using a Grating-Coupling Surface Plasmon Resonance (GC-SPR) system [28,29,30].

The 15-mer PNA used here is complementary to a target sequence in genomic DNA of *Mycobacterium tuberculosis*. The development of a sensor for *M. tuberculosis* detection based on Surface Plasmon Resonance could respond to the current need for a sensitive, specific, fast and cost-effective method for detection of this pathogen that, causing tuberculosis, is the cause of death of nearly three million people every year [31].

## 2. Results and Discussion

### 2.1. Probe Design

The structure of the designed probe is reported in Scheme 1. It is composed of three main elements, namely a thiol function for gold attachment, the high MW PEO chain meant to prevent non-specific interactions and a sequence for DNA recognition. Since thiols are highly sensitive to oxidation, a chemical protection is generally recommended, which needs to be removed before gold anchorage. For protection, we selected the trityl chemistry since it can be easily removed by 5 min acidic treatment with TFA, and the leaving group (trityl cation) does not have affinity for gold, as opposed to the thiolated protecting elements used in the asymmetric disulfide protection strategy. With respect to the DNA binding element, a PNA analogue was selected on the basis of its ability to improve duplex stability and base mismatch discrimination.

### 2.2. Selection of the PNA Sequence and Length

We selected a 15 bases long probe within an 81-bp fragment of the gene encoding the beta subunit of RNA polymerase (RpoB) whose mutations are involved in rifampicin resistance [32]. The probe (RPOB15wt) was analyzed by BLAST to avoid high homology with other genomes. We have selected a 15 base length probe as a compromise between affinity and specificity for the target analyte. In fact, oligomers of 12 to 17 bases are suitable for most applications whereas longer sequences (from 25 to 40 units) are rarely required to improve the stability of the hybridized duplex. In addition, for the discrimination of single base mutations as those that generate drug resistance, the impact of a mismatch is greater on a shorter sequence than a longer one. It is generally accepted that relatively short sequences (less than 15 residues) can discriminate single base mismatch through hybridization at room temperature with appropriate control of salt concentration [33]. On the other hand, if longer probes are used, salt concentration modification may not be sufficient to destabilize unpaired duplex for single mismatch discrimination and higher temperature or the addition of denaturants such as formamide must be used, and both the use of high temperature or formamide may not be feasible within common SPR experimental set-ups [11,33,34].

From the chemical point of view it has to be pointed out that purine-rich PNA oligomers display a tendency to aggregate, with guanine-rich oligomers having the greatest tendency [35]. As a consequence, the selected 15-mer (PNA-RPOB15wt) can be considered a “difficult” one to synthesize because it includes more than three guanine (G) residues in a row and it also includes more than six purines in any stretch of ten units. In addition, the sequence contains a palindrome trait (GCGC) that could potentially give problems such as self-hybridization with consequent difficulties in purification and characterization.

### 2.3. Synthesis and Characterization of PNA-RPOB15wt

Due to the predicted difficulties in the synthesis of the selected sequence, PNA elongation on the solid support was carefully monitored with sampling of resin at different stages of the PNA chain growth. In addition, special attention has been paid in the last six elongation steps: 10 eq. of PNA monomer were used for the condensation of the 11th base and double couplings (with 5 eq. of PNA monomer each) were carried out for the last four insertions. Mass data confirmed that, at each step, the principal compound in the crude mixture was the target product. In the mass spectrum of 10-mer PNA, a signal at 2717.06 Da revealed the presence of acetylated 9-mer PNA, suggesting to introduce a double coupling to increase the reaction yield after the 9th oligomer insertion. (see Supplementary Information).

**Figure 1:**
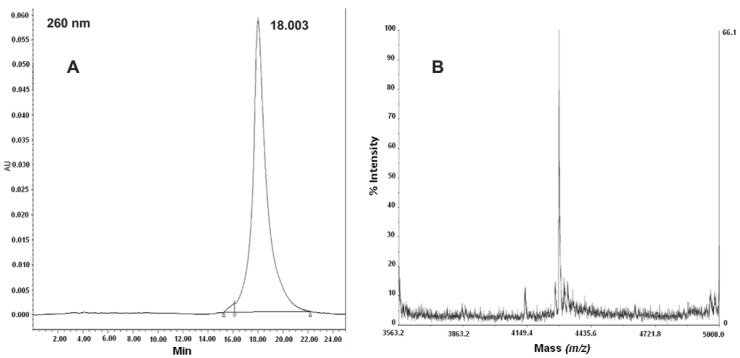
(**A**) RP-HPLC of purified PNA-RPOB15wt. Conditions used: Vydac analytic C18 monomeric 238TP54 (5 μm, 300 Å, 4.6 × 250 mm); (**B**) Deconvoluted ESI-TOF mass spectrum of the purified PNA-RPOB15wt. (experimental mass = 4292.7 Da; theoretical mass = 4292.94 Da).

Purification of the final product from the crude mixture was carried out by semipreparative reverse phase column. In order to limit aggregation processes, a very dilute crude PNA solution (about 10 mg crude PNA in 30 mL 0.05% TFA/MilliQ water) was prepared, which was slowly loaded onto the reverse phase medium. The chromatogram of the purified product and the electrospray mass spectrum data are reported in Figure 1. The final yield in purified product was 49.89%.

### 2.4. Solid-Phase Conjugation of Fmoc-Cys(Trt)-PEO_5KDa_-COOH to PNA

Solid phase modification of matrix-bound sequences is a convenient procedure in peptide or PNA synthesis. It is easy to perform and it allows fast purification from the excess of coupling reagents. However, when it comes to linking relatively large moieties, the efficiency of this method suffers from steric limitations, namely a reduced accessibility for the bulky reactive carboxyl to the matrix-bound peptide (or PNA) amines. Nevertheless, we still attempted solid phase PEO coupling, mainly to compare its efficiency with that of the solution approaches. Condensation was carried out using 10 eq. excess of Fmoc-Cys(Trt)-PEO_5KDa_-COOH with respect to PNA, that is the same conditions as those adopted in peptide synthesis. Reaction yield was calculated from the absorbance generated by the matrix tethered Fmoc group (after piperidine treatment). The yield was only 5.22%, confirming the impracticability of this strategy, at least in the proposed experimental conditions (solvent, type of reagents, type of resin, temperature, *etc.*).

### 2.5. Conjugation of Fmoc-Cys(Trt)-PEO_5KDa_-COOH to PNA in Solution

One of the drawbacks of both PNA and PEO technologies is the high cost of both classes of compounds. Therefore, we paid particular attention to the reagents’ relative molar ratio, with the general purpose to avoid large excess of any of them, thus minimizing waste and process costs. 

Two synthetic strategies were attempted, using two known condensing agents, namely *O*-(7-azabenzotriazol-1-yl)-*N,N,N' ,N'*-tetramethyluronium hexafluorophosphate (HATU) and [ethyl cyano (hydroxyimino)acetate-O_2_] tri-1-pyrrolidinylphosphonium hexafluorophosphate (PyOxim). HATU is the most common coupling agent used for amidation. PyOxim is a new coupling reagent more efficient than HATU that mediates coupling with low racemisation or epimerisation and is ideal for fragment condensation because it cannot cause guanidinylation [36].

The solubility of PNA-RPOB15wt (henceforth also called “PNA”) in different organic solvents was measured in order to select the most suitable environment for the solution coupling reaction. Based on the results obtained (Table S-1 supplementary information) dimethylsulfoxide (DMSO) was selected.

All reactions were carried out using purified PNA at about 5 mM concentration adding 2 eq. of Fmoc-Cys(Trt)-PEO_5KDa_-COOH. Reactions were followed by measuring the decrease of primary amines in solution using the sensitive and fast fluorescamine test [37]. Condensing agents were added by multiple additions (Table 1) and free amines in solution were measured at scheduled times after each addition, until no fluorescamine reading was reached.

**Table 1:** Equivalents of coupling reagents used for PNA-PEO solution coupling and degree of amine conversion obtained.

Eq. ratio HATU/DMAP	% of amine modification	Eq. ratio PyOxim:TEA	% of amine modification
1	0	10:2	0
10	44	10:10	33
30	90	10:20	52.1
36	100	20:20	72.3
		40:30	100

Thirty-six equivalents of HATU/DMAP were necessary to achieve full amine conversion in 2 h. The PyOxim catalyzed reaction was stopped after the addition of 40 and 30 equivalents of PyOxim and TEA respectively, that lead to quantitative amine conversion.

### 2.6. Isolation of PNA-PEO from Crude Reaction Mixture and Chromatographic Characterization

The components of the two crude reaction mixtures were isolated by diethyl ether precipitation. This procedure yields to the co-precipitation of all compounds in solution so that the powders obtained are composed of the desired products together with the coupling reagents and the excess of PEO derivative. Among all of these impurities, the excess of PEO reagent, that contains thiols capable to react with gold and compete with the PNA-PEO conjugate, is the most important to remove. 

**Figure 2:**
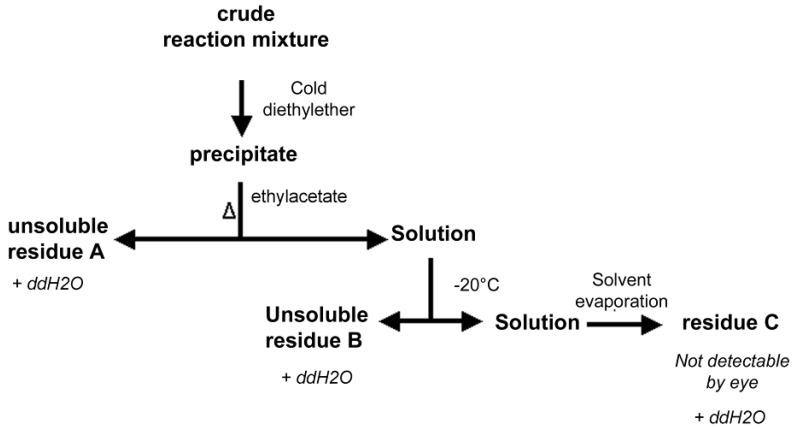
The differential solvent extraction process adopted.

The possibility to purify the PNA-PEO conjugate from the crude reaction mixture by means of a differential solvent extraction protocol was investigated. The process adopted is summarized in Figure 2. Rationale for designing this protocol was the known solubility of PNA and PEO in different organic solvents: PEO is insoluble in cold ethyl acetate but soluble in the same after heating. It is also extremely soluble in aqueous solutions. PNAs are not soluble in neither diethyl ether, ethyl acetate nor aqueous buffer (Table 1). The process depicted in Figure 2 was conducted to verify if hot ethyl acetate treatment could efficiently separate the starting PEO derivative from its PNA conjugated form. 

**Figure 3:**
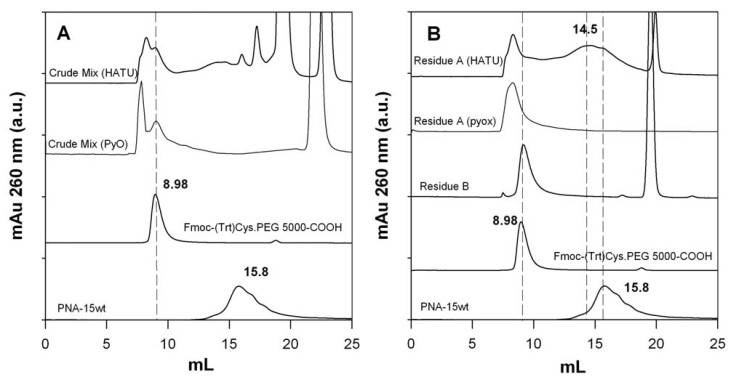
Gel permeation chromatography of crude reaction mixtures (**A**) and residues A and B (**B**) obtained with the HATU/DMAP and PyOxim/TEA coupling reagents. The elution profiles of non modified PNA and the PEO reagent are also shown for reference.

As a result of the process of Figure 2, we obtained three samples, named residues A, B and C. Since the solubility properties of the desired conjugate were unknown, all three residues were dissolved in water and were extensively analyzed, first by UV-Vis spectrometry, then if UV_260nm_ positive (indication of potential PNA presence), by gel permeation chromatography (GPC). Figure 3 shows the size exclusion chromatograms of the crude mixture before diethyl ether precipitation together with residues A and B (the only ones that were UV_260nm_ positive). The GPC profiles of the two starting reagents (PNA and PEO derivative) are also shown for reference. 

The starting Fmoc-Cys(Trt)-PEO_5KDa_-COOH and native PNA derivative elute at 8.98 and 15.8 mL, respectively. Interestingly, the native PNA retention volume is of the same order of that expected for lower MW compounds and indicates the existence of a chemical interaction with the Superdex peptide™ matrix This is not new for polysaccharide-based gel chromatography media [38,39] and, in this case, we suppose it to be due to the columns dextran component only, since a pure agarose medium (Superose™) did not show a similar deviation from size exclusion behavior (data not shown).

The reaction crudes (before ether precipitation) show a number of peaks that elute along the entire column separating capability. The compounds eluting at the end of the run correspond to the low molecular weight HATU and DMAP (20 mL) and DMSO (22 mL). These low molecular weigh components were removed along the diethyl ether precipitation and ethyl acetate extraction.

**Figure 4:**
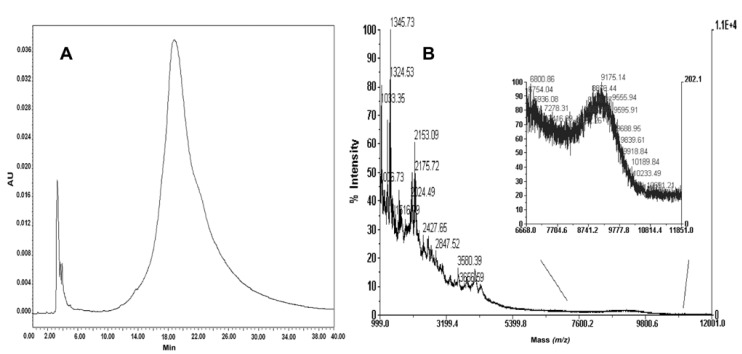
(**A**) RP-HPLC and (**B**) MALDI analysis of residue A PNA-PEG_5KDa_ conjugate product obtained through PyOxim/TEA coupling (experimental mass = 9,175 Da; theoretical mass = 9,430 Da).

The main crude components elute with a double peak centered at 8.28 and 8.98 mL, the first one corresponding to the desired product and second one corresponding to excess of PEO reagent. The latter was quantitatively extracted by ethyl acetate treatment, as demonstrated by the chromatograms of residues A and B. In the case of PyOxim/TEA coupling, the desired conjugate (8.28 mL) appears as the only conjugation product, as confirmed by RP-HPLC and by MALDI analyses (Figure 4). 

MALDI analysis clearly shows the presence of the conjugate, event if the intensity of its peak is low and is ‘masked’ by a number of other peaks at low *m/z* values. We believe that this pattern is not related to the purity of the product, but most likely accounts for its low ionization efficiency, particularly due the presence of the high MW PEO moiety that causes signal dispersion. Indeed, it has to be pointed out that ionization of this kind of conjugates requires a laser power higher than the one that is usually applied for the analysis of other species such as peptides or simple PNAs. The enhanced laser power induces the formation of matrix clusters that create artefact peaks in the low *m/z* spectral range. Most importantly, the occurrence of even trace amounts from prompt in-source fragmentation induces strong ion suppression effects, resulting in hardly detectable MS signals of the PEO-PNA conjugate.

In the case of HATU/DMAP coupling FPLC analysis revealed the presence of an additional broad peak, centered at 14.5 mL, whose nature is unclear. Since no primary amines were left after coupling (Table 1), we hypothesize that it is a side product deriving from an undesired side reaction between PNA and the condensing agent HATU. Indeed, as reported in literature [40], the use of excess of HATU with respect to the carboxylic group bearing reagent can favor a secondary reaction between the amino bearing reagent and HATU itself with the consequent locking of amino functional group (guanidinylation).

**Figure 5:**
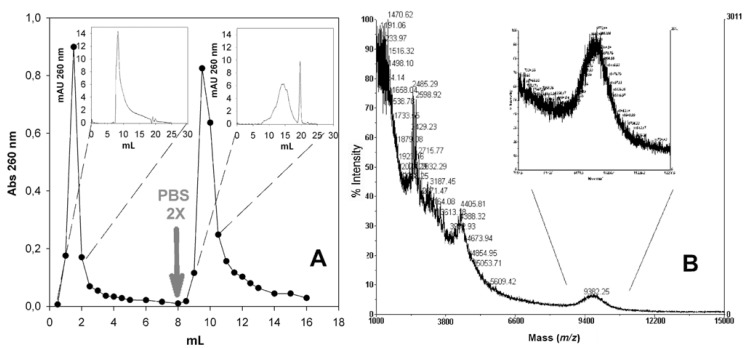
(**A**) NAP5 elution profile of Residue A of the HATU/DMAP reaction and FPLC analysis the two NAP5 peaks; (**B**) MALDI spectrum of the Sephadex G25 (NAP5) fraction eluted using H_2_O as eluent.

We explored the possibility to further purify the desired product from high retention volume impurities by taking advantage of the hypothesized affinity of non-PEGylated PNA for the Superdex peptide dextran component. In order to verify this hypothesis and try to further take advantage of it, residue A was eluted through a pure dextran gel permeation medium, namely Sephadex G25, using a gravity column (NAP5). In order to maximize the polar forces responsible for the affinity between PNA and the column, the latter was equilibrated and initially eluted with dd-H_2_O, followed by the higher ionic strength buffer PBS 2X. The main UV positive fractions eluted in H_2_O and buffer, were collected and analyzed by FPLC and MALDI. Figure 5A shows the NAP5 elution profile and FPLC chromatograms of the eluted peaks, proving the successful separation of the two residue A major components. MALDI analysis (Figure 5B) confirmed the presence of the PEGylated conjugate in the H_2_O eluted fraction only.

Reaction yields were calculated by the combination of UV-Vis spectrophotometry and chromatography data. The amount of PNA recovered after ethyl acetate treatment was 90%–94.6% of the starting PNA. The degree of PNA conversion into the PEGylated derivative was 49.6% and 100% for the DMAP/HATU and PyOxim/TEA couplings respectively.

### 2.7. Conjugation of Fmoc-Cys(Trt)-PEO_2KDa_-COOH to PNA

Conjugation with the 2 KDa PEO derivative was carried out on a smaller scale (1 mg of PNA) using the DMAP/HATU coupling reagents only, obtaining similar results as those with the 5 KDa PEO (see Supplementary Information).

### 2.8. Application of the PNA-PEO Conjugate to SPR Detection

**Figure 6:**
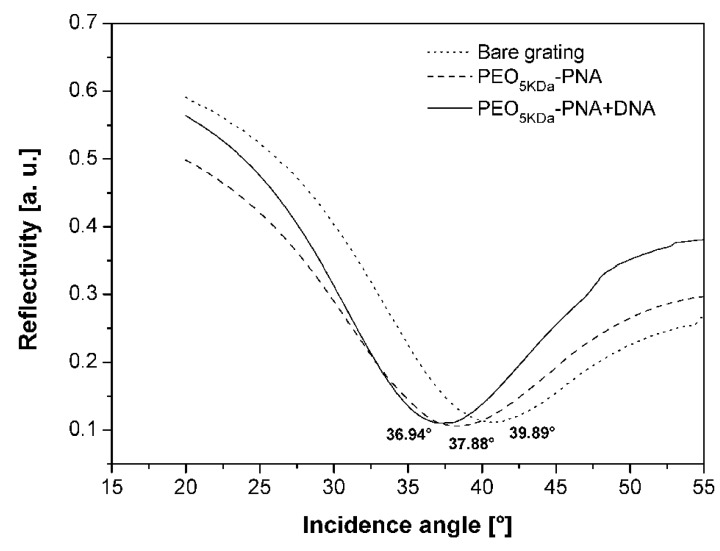
Reflectivity curves obtained on the bare grating as such, after PNA-PEO_5KDa_ grafting and complementary DNA hybridization.

In order the evaluate the efficacy of the synthesized bioconjugates to work as probes for DNA sensing, we performed SPR Grating-Coupling Surface Plasmon Resonance (GC-SPR) measurements. These measurements are carried out using a device that has a geometry alternative to the classic Kretschmann-Raether one, which permits bypassing the use of the prism [28,29,30]. This is achieved by substituting the flat sensing surface with a nanopatterned sinusoidal one. In this type of SPR device, successful formation of a dielectric layer, such as that generated upon PEO-PNA deposition and, later, DNA/PNA hybridization, causes a shift in the SP resonance angle, due to a variation of the dielectric surrounding medium refractive index (which is related to the amount of material grafted to the surface). Reflectivity shift measurements were therefore carried out on the gold substrate as such, after functionalisation with the PNA-PEO_5KDa_ conjugate and after DNA hybridization. Figure 6 shows the reflectivity dips obtained for the bare grating, after Fmoc-Cys-PEO_5KDa_ PNA functionalisation and after complementary DNA hybridization. The SPR angle shifts were 2.01 ± 0.05° and 0.94 ± 0.04° for the first and second step respectively, demonstrating the ability of the synthetic probe to both tether the gold surface and to recognize its target. Experiments on sensitivity and selectivity of the sensing strategy adopted are currently underway.

## 3. Experimental

### 3.1. Materials

Tentagel Sieber Amide resin was purchased from Rapp Polymer (Tuebingen, Germany). Fmoc-Ala-OH was from Novabiochem Merck (Darmstadt, Germany), whereas Fmoc protected PNA monomers were purchased from Polyorg (Leominster, MA, USA); α-amino-ω-carboxy-poly(ethylene glycol) (H_2_N-PEO-COOH, 2 KDa and 5 KDa) was purchased from Laysan Bio (Huntsville, AL, USA), or IRIS Biotech Gmbh (Marktredwitz, Germany), 2-(1*H*-7-azabenzotriazol-1-yl)-1,1,3,3-tetramethyl uronium hexafluorophosphate (HATU) and *N*-hydroxybenzotriazole (HOBt) were purchased from Advanced Biotech Italia (Milan, Italy). *N*-hydroxysuccinimide (NHS) and diciclohexylcarbodiimide (DCC) were purchased from ABCR (Karlsruhe, Germany). *N,N-*dimethylformamide (DMF), *N*-methylpyrrolidone (NMP), 2,2,2-trifluoroacetic acid (TFA) and *N,N-*diisopropylethylamine (DIPEA) were purchased by Biosolve (Valkenswaard, The Netherlands). Diethyl ether, dichloromethane (DCM), ethyl acetate, acetonitrile (CH_3_CN), 2,6-lutidine, acetic anhydride (Ac_2_O), methanol (MeOH), 4-dimethylaminopyridine (DMAP), triethanolamine (TEA), PyOxim (PyO) and all other chemicals were from Sigma-Aldrich (St. Louis, MO, USA). Water was of MilliQ (Millipore, Billerica, MA, USA) or double distilled (dd-H_2_O) grade. Amino-containing compounds were detected using ninhydrin [41,42] and/or quantified in solution either by the trinitrobenzenesulfonic acid assay (TNBS) [43,44] or with fluorescamine [37]. PEO in solution was detected and quantified by the iodine reagent [44,45]. Thiols in solution were titrated using the 5,5'-dithio-bis-(2-nitrobenzoic acid) (DTNB) reagent [46]. Solid-phase synthesis was carried out using an automated peptide synthesizer, Syro I (MultiSynTech GmbH, Witten, Germany). NMR analyses were carried out on a Bruker (Madison, WI, USA) AMX 300 MHz instrument; UV spectra were recorded on a Cary 50 UV-Vis spectrophotometer (Varian-Agilent, Santa Clara, CA, USA). Fluorescence was determined using a JASCO (Great Dunmow, Essex, UK) FP-6200 spectro-fluorimeter. Fast Permeation Liquid Chromatography (FPLC) analyses were performed using an AKTA purifier 10 (GE Healthcare, Uppsala, Sweden) integrated with a UV-Vis detector and refractive index analyzer (Waters Corporation, Milford, MA, USA). RP-HPLC (reverse-phase-HPLC) was performed using a Waters 600E System Controller equipped with a UV/Vis detector Mod. 2487. Mass analyses were carried out using an ESI-TOF Applied Biosystems Mariner System 5220 (Perkin-Elmer, Waltham, MA, USA) or a MALDI-TOF Voyager DE-Pro (Perseptive Biosystem, Framingham, MA, USA) instrument, utilizing 2,5-dihydroxybenzoic acid (DHB), dissolved in 10 mg/mL in 50% acetonitrile (v/v), containing 0.1% TFA, as the matrix. Thiolene resin (NOA 61) was purchased from Norland Optical Adhesives (Cranbury, NJ, USA). Complementary DNA (5'-CCCCAGCGCCGACAG-3') was purchased from the Sigma Aldrich custom synthesis service.

### 3.2. Synthesis of Fmoc-Cys(Trt)-PEO_2KDa_-COOH and Fmoc-Cys(Trt)-PEO_5KDa_-COOH

**Scheme 2:**
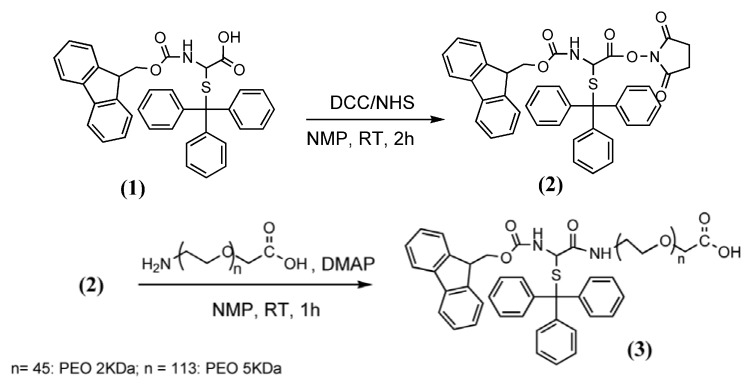
Synthesis of Fmoc-Cys(Trt)-PEO_5KDa_-COOH (**3**).

The same protocol was used to synthesize both 2 and 5 KDa derivatives (Scheme 2). Fmoc-Cys(Trt)-OH (**1**) (500 mg, 0.85 mmol) was activated with NHS (104 mg, 1.0 eq.) and DCC (175 mg, 1.0 eq.) in dry NMP (15 mL). The reaction was followed by thin layer chromatography (TLC, CHCl_3_–MeOH = 40:10). After 2 h, the solution containing the activated product **2** was Gooch-filtered and 5.3 mL (0.3 mmol of **2**) were immediately added dropwise to 0.2 mmol of NH_2_-PEO-COOH HCl dissolved in anhydrous NMP (50 mL). 

Reaction completion was verified through the ninhydrin test. The product **3** was precipitated with dry diethyl ether and isolated by Gooch filtration. Purification from excess of **1** and reaction side-products was achieved by re-dissolving the powder product in NMP and precipitation in dry diethyl ether. The degree of PEO amine modification was measured by the TNBS assay [43,44]. The yield of α-amino conversion was 95% for both PEO derivatives. Products were also analyzed by gel permeation chromatography and MALDI mass spectrometry (see Supplementary Information). Gel permeation analysis was also carried out using a Superdex peptide 10/300 GL column; loop 100 μL, T = room temperature, eluent: PBS buffer, flow rate: 0.5 mL/min, detector: readings at 260 nm. Yield: 560 mg (56%) for PEO_5KDa_ and 340 mg (85%) for PEO_2KDa_.

### 3.3. PNA Synthesis

The PNA RPOB15wt (5'-CTGTCGGCGCTGGGG-Ala-Ala-NH_2_) was synthesized by adaptation of solid-phase peptide synthesis protocols. The synthesis was carried out using 0.020 mmol Tentagel Sieber Amide resin (0.2 mmol/g) and 5 eq. of Fmoc amino acids or Fmoc-PNA monomers (0.1 mmol) for each condensation. The following PNA monomers were used: *N*-(*N*-Fmoc-2-aminoethyl)-*N*-[(*N*-4-Bhoc-1-cytosyl)acetyl]-glycine, *N*-(*N*-Fmoc-2-aminoethyl)-*N*-[(*N*-6-Bhoc-9-guanyl)acetyl]-glycine, *N*-(*N*-Fmoc-2-aminoethyl)-*N*-[(1-thyminyl)acetyl]-glycine.

#### 3.3.1. Condensation of Amino Acids

Fmoc deprotection of the resin or the amino acids was obtained by two treatments: the first one consisting in 40% piperidine in DMF for 3 minutes (min), and the second one consisting in 20% piperidine in DMF for 12 min. Loading of the first two amino acids was carried out with double couplings. Each coupling was carried out by activating 0.1 mmol of Fmoc-Ala-OH (5 eq.) in DMF (500 μL) with 0.2 M HATU/HOBt (5 eq.) in DMF (500 μL) and 0.4 M DIPEA (5 eq.) in NMP (500 μL) for 45 min. After the second insertion reaction, the yield was determined by measuring the absorbance of *N-*(9-fluorenylmethyl)piperidine at 301 nm and resulted 97.62%.

#### 3.3.2. Condensation of PNA Monomers

Removal of the Fmoc group was obtained by two treatments, of 30 s each, with 20% piperidine in DMF; couplings were carried out activating 0.1 mmol of Fmoc-protected PNA monomers (5 eq.) in NMP (500 μL) with 0.2 M HATU/HOBt in DMF (500 μL) and 0.2 M DIPEA/0.3 M lutidine (5 eq./7.5 eq.) in DMF (500 μL). Reaction time was 45 min for single couplings and 90 min (2 × 45) for double couplings. Finally a capping reaction was performed adding 1 mL of 5% Ac_2_O/6% lutidine in DMF for 3 min.

The first ten PNA monomers were introduced with single couplings, using 5 eq. of Fmoc-protected PNA monomer; the 11th monomer was introduced by single coupling using 10 eq. of activated monomer, whereas the remaining four insertions were done by double couplings (each using 5 eq.). At the end of the synthesis, the *N-*terminal Fmoc protecting group was removed; the resin was washed 6 times with DMF and 3 times with DCM and then dried under vacuum for 1 h. Finally, PNA cleavage from the resin and protecting groups de-blocking was obtained using TFA-MilliQ water-triethylsilane (TES) = 90:5:5 for 10 min under slow manual stirring. The solution was concentrated to small volume and crude PNA was precipitated with cold diethyl ether under magnetic stirring, isolated by centrifugation and then dried under vacuum for 20 min.

We monitored the synthesis of PNA RPOB15wt by controlling the partial products obtained after the introduction of single monomers starting from the 10th insertion. To this end, small aliquots of each intermediate product (10-mer or 11-mer, 12-mer, 13-mer, 14-mer and 15-mer) was detached from the resin and the protecting groups were removed as previously described; crude products were characterized by mass spectrometry and by RP-HPLC using a Vydac analytic C18 monomeric 238TP54 column (5 μm, 300 Å, 4.6 × 250 mm, Grace, Columbia, MD, USA). All partial product identity was confirmed by mass spectrometry using ESI-TOF system: 10-mer (theoretical value: 2966.70 Da; experimental value: 2966.16 Da); 11-mer (theor.: 3217.93 Da; exp.: 3217.42 Da); 12-mer (theor.: 3484.19 Da; exp.: 3484.36 Da); 13-mer (theor.: 3775.45 Da; exp.: 3775.42 Da); 14-mer (theor.: 4041.71 Da; exp.: 4041.70 Da); 15-mer (theor.: 4292.94 Da; exp.: 4292.70 Da). All HPLC chromatograms and mass spectra are reported in the Supplementary Information section.

Purification of the final product from crude was performed by RP-HPLC using a Delta Pack C18 semipreparative column (6 μm, 60 Å, 7.8 × 300 mm, Waters Corporation); T = 55.5 °C; eluent A, 0.05% TFA/MilliQ water; eluent B, 0.05% TFA/CH_3_CN; gradient, 10 min at 8% of B and from 8% to 35% of B over 27 min; detector, 214 nm and 260 nm; flow rate, 4 mL/min. The purity of the collected final product was verified by RP-HPLC using the Vydac analytic C18 monomeric 238TP54 column (5 μm, 300 Å, 4.6 × 250 mm); T = 55.5 °C; eluent A, 0.05% TFA/MilliQ water; eluent B, 0.05% TFA/CH_3_CN; gradient, from 5% to 55% of B over 25 min; detector, 260 nm (purity grade = 98.96%); flow rate, 1 mL/min.

### 3.4. Solid-Phase Conjugation of Fmoc-Cys(Trt)-PEO_5KDa_-COOH to PNA

Fmoc-deprotected PNA RPOB15wt (46.60 mg, 4.03 μmol, henceforth referred to as PNA) on resin were swelled in DMF (1 mL) for 1 h. After DMF drain, the PNA-resin was added with Fmoc-Cys(Trt)-PEO_5KDa_-COOH (**3**, 20 μmol), dissolved in DMF (500 μL), 20 μmoles of 0.3 M DIPEA/0.2 M lutidine in DMF (100 μL), 20 μmol HATU/HOBt 0.2 M in DMF (100 μL). After 23 h at room temperature, the mixture was separated by filtration and the resin was washed three times with DMF, and three times with DCM and then dried under vacuum for 1 h. The solution was concentrated to small volume and the unreacted Fmoc-Cys(Trt)-PEO_5KDa_-COOH was recovered by cold diethyl ether precipitation followed by filtration. Reaction yield was determined by treating a small aliquot of the resin with piperidine and measuring Fmoc-piperidine adduct at 301 nm. 

Detachment of the PEO-PNA conjugate from the resin together with removal of the side chain protecting groups was carried out adding a TFA-MilliQ water-TES-1,2-ethanedithiol (EDT) (94:2.5:2.5:1) solution and m-cresol (m-cresol:TFA = 1:4) to the PEO-PNA-resin for 30 min under slow manual stirring. The solution was concentrated to small volume and the product was precipitated with cold diethyl ether under magnetic stirring, isolated by centrifugation and then dried under vacuum for 1 h. It was then analyzed by gel permeation chromatography using an AKTA purifier Fast Permeation Liquid Chromatography (FPLC) system integrated with an UV-Vis and a refractive index detectors. Analyses were performed using a Superdex peptide 10/300 GL (Superdex peptide) column.

### 3.5. Solution Conjugation of Fmoc-Cys(Trt)-PEO-COOH to PNA

#### 3.5.1. HATU/DMAP as Coupling Reagents

This reaction was carried out using both 2 and 5 KDa PEO derivatives. Reaction with Fmoc-Cys(Trt)-PEO_5KDa_-COOH was carried out on 15 mg of PNA, whereas the reaction with Fmoc-Cys(Trt)-PEO_2KDa_-COOH was carried out on a smaller scale (1 mg). Coupling was carried out in dry DMSO. Stock solutions containing both PNA and Fmoc-Cys(Trt)-PEO_5KDa_-COOH (or Fmoc-Cys(Trt)-PEO_2KDa_-COOH) in dry DMSO were prepared at 5 mM and 10 mM concentration, respectively. One M stock solutions of DMAP and HATU in DMSO were prepared separately and later added in equal volumes to the PNA/PEO until the final PNA:HATU:DMAP ratio was reached. Mixtures were gently stirred at room temperature and small aliquots (10 μL) were removed at scheduled times for primary amine determination by the fluoresamine assay [37]. Analyses were carried out 2 h after each addition. The reaction was stopped by cold diethyl ether precipitation (20 volumes with respect to the reaction mixture) when the reaction solution was negative to the fluoresamine test.

#### 3.5.2. PyO/TEA as Coupling Reagents

This reaction was carried out using the 5 KDa PEO derivative only, using 3 mg of PNA. The initial PNA and Fmoc-Cys(Trt)-PEO_5KDa_-COOH mixture in dry DMSO was prepared as above at 5 and 10 mM concentration respectively. One M TEA and PyO stock solutions in dry DMSO were prepared and added, separately, in small portions to the PNA/PEO mix. After each addition, aliquots of the reaction mixture (10 μL) were removed for primary amine determination by the fluorescamine assay [37]. Analyses were carried out 1 h after each addition. The reaction was stopped as described above. 

### 3.6. PNA-PEO Purification from Crude Mixture

The diethyl ether solid white precipitates were isolated by centrifugation at 4 °C (16,600 *g*, 5 min). They were then added of ethyl acetate (same volume as that of diethyl ether) and heated at 60 °C for 2 min, then centrifuged at 25 °C (16,600 *g*, 37 °C, 5 min). The solid was isolated (residue A) and the clear ethyl acetate solution was cooled overnight at –21 °C. Upon ethyl acetate cooling, a second white precipitate was obtained (residue B), which was isolated by centrifugation (16,600 *g*, 5 min, 4 °C). The clear ethyl acetate solution was finally evaporated to dryness leading to residue C. 

Residues A, B and C were dissolved in H_2_O and analyzed by UV-Vis. The 260 nm UV positive samples were analyzed by MALDI mass spectrometry and gel permeation chromatography. Chromatography analysis was performed using the AKTA purifier chromatography system and a Superdex peptide 10/300 GL column. In some cases, for peak identification, the eluting solutions were fraction-collected and the individual fractions (0.5 mL) were analyzed by UV-Vis spectrophotometry and by the iodine assay to measure PEO concentration [45]. 

Residue A from the HATU/DMAP reaction was further purified by gel exclusion chromatography using a dextran (Sephadex) gravity column (NAP5, GE, Amersham Pharmacia Biotech, Uppsla, Sweden). The sample was dissolved and eluted in dd-H_2_O, first, followed by 20 mM sodium phosphate, 300 mM NaCl, pH 7.4 (PBS 2X). Fractions (250–500 μL) were collected and analyzed by UV-Vis spectrometry and gel permeation chromatography.

### 3.7. SPR Measurements

SPR reflectivity measurements were performed on a J. A. Woollam Co. VASE ellipsometer with angular and wavelength spectroscopic resolution of 0.005° and 0.3 nm respectively. The azimuthally rotated (φ = 45° and α = 140°) detection configuration was adopted performing an angular scan from 20° to 80° at the incident wavelength of 625 nm. 

SPR substrate (period of 500 nm and amplitude of 40 nm) was obtained by the replica of a grating fabricated through Laser Interference Lithography [28] onto a thiolene resin (NOA 61) film. The surface was then coated with a bi-metallic Cr (5 nm)/Au (40 nm) layer. The substrate was cleaned (10 min in a 5:1:1 dd-H_2_O, NH_4_OH and H_2_O_2_ solution) immediately prior to functionalisation.

Probe grafting was achieved after removal of the Trt-protective group by dissolving the PNA- PEG_5KDa_ powder in the minimum amount of TFA for 20 min at room temperature, then diluted in dd-H_2_O volume at final 1mM concentration. The insoluble Trt residue was removed by centrifugation (10,000 *g*, 4 °C, 10 min). The substrate was immersed overnight into the clear supernatant solution at 30 °C, then rinsed dd-H_2_O to remove physisorbed molecules, and dried under vacuum for 30 min. 

DNA hybridization was achieved by incubating the probe functionalized substrate with the complementary sequence 0.1 µM in 150 mM NaCl, 15 mM Na citrate, pH 7 (SCC) buffer, for 2 h at 30 °C. Before SPR measurement, the substrate was rinsed in SSC and dried under nitrogen flux. The exact dip resonance angle at each point was determined by fitting the reflectivity curves with a Lorentzian function and using the centroid value as reference.

## 4. Conclusions

The PNA-PEO coupling reaction proved to be sterically hindered as demonstrated by the large excess of condensing agents necessary to achieve quantitative PNA derivatization. The use of HATU lead to only 49.6% and 5.2% conversion yields, in solution and solid phase respectively, and to the formation of a non-PEGylated amino-modified PNA as side product. Coupling with PyOxim was much more efficient, leading to 100% conversion into the desired conjugate, without generating undesired side products. Only two equivalents of bi-functional PEOs were used in all couplings and the excess was successfully removed by a convenient chromatography-free solvent extraction procedure. Purification from the latter is fundamental for application since this molecule would compete with the PNA conjugate for surface functionalisation. 

The combination of the PyOxim-based solution coupling and the purification process here optimized is convenient and economical, and provides the product in a mixture that can be used as such for surface functionalisation. The Sephadex gravity purification step, necessary after the HATU/DMAP coupling procedure only, is also convenient and economical. 

The 5 KDa conjugate was successfully anchored to gold and was capable to act as a probe for complementary DNA recognition, demonstrating the ability of the synthetic probe to both tether a gold surface and to recognize its target. Experiments on sensitivity and selectivity of the SPR sensing strategy adopted are currently underway. 

As a final statement, we would like to underline that the PEO coupling strategy here devised is applicable to any amino-PNA and is also reproducible in laboratories that are not highly equipped for chemical synthesis. We hope this will permit expanding the use of this kind of bioconjugates for biosensing and/or other biomedical application.

## Supplementary Information

In this section, we report the RP-chromatrograms (Figure S-1) and mass spectra (Figures S-2 to S-7) of the intermediate crude products of PNA: 10-mer, 11-mer, 12-mer, 13-mer, 14-mer and 15-mer. The solubility of the 15mer PNA in different solvents is reported in Table S-1. The MALDI spectra of Fmoc-Cys(Trt)-PEO_2KDa_-COOH and of Fmoc-Cys(Trt)-PEO_5KDa_-COOH are reported in Figure S-8. The gel permeation profiles in superdex peptide column of the above products are shown in Figure S-9. The results of the coupling using the 2 KDa PEO derivative are summarized in Figure S-10.

Supplementary materials can be accessed at: http://www.mdpi.com/1420-3049/17/9/11026/s1.
