# Supplementary material for: Synthesis and Chromatography-Free Purification of PNA-PEO Conjugates for the Functionalisation of Gold Sensors
Source: Molecules. 2012 Sep 13;17(9):11026–45. doi: 10.3390/molecules170911026 (PMC6268724; doi:10.3390/molecules170911026)

# Supplementary Information

## 1. RP-HPLC Analyses and Mass Spectra of the Intermediate Crude Products of PNA-RPOB15wt

We reported here the RP-HPLC analyses (Figure S-1) and mass spectra and deconvolution of all the intermediate crude products of PNA-RPOB15wt: 10-mer (Figure S-2), 11-mer (Figure S-3), 12-mer (Figure S-4), 13-mer (Figure S-5), 14-mer (Figure S-6), and 15-mer (Figure S-7).

**Figure S-1.** Overlay of RP-HPLC of crude PNA 10-mer (red), 11-mer (blue), 12-mer (magenta), 13-mer (green) and 15-mer (black). Conditions used: Vydac analytic C18 monomeric 238TP54 (5 µm, 300 Å, 4.6 × 250 mm); T = 55.5 °C; eluent A, 0.05% TFA/MilliQ water; eluent B, 0.05% TFA/$CH_3CN$; gradient, 5 min at 0% of B and from 0 to 100% of B over 25 min; detector, 260 nm; flow rate, 1 mL/min.

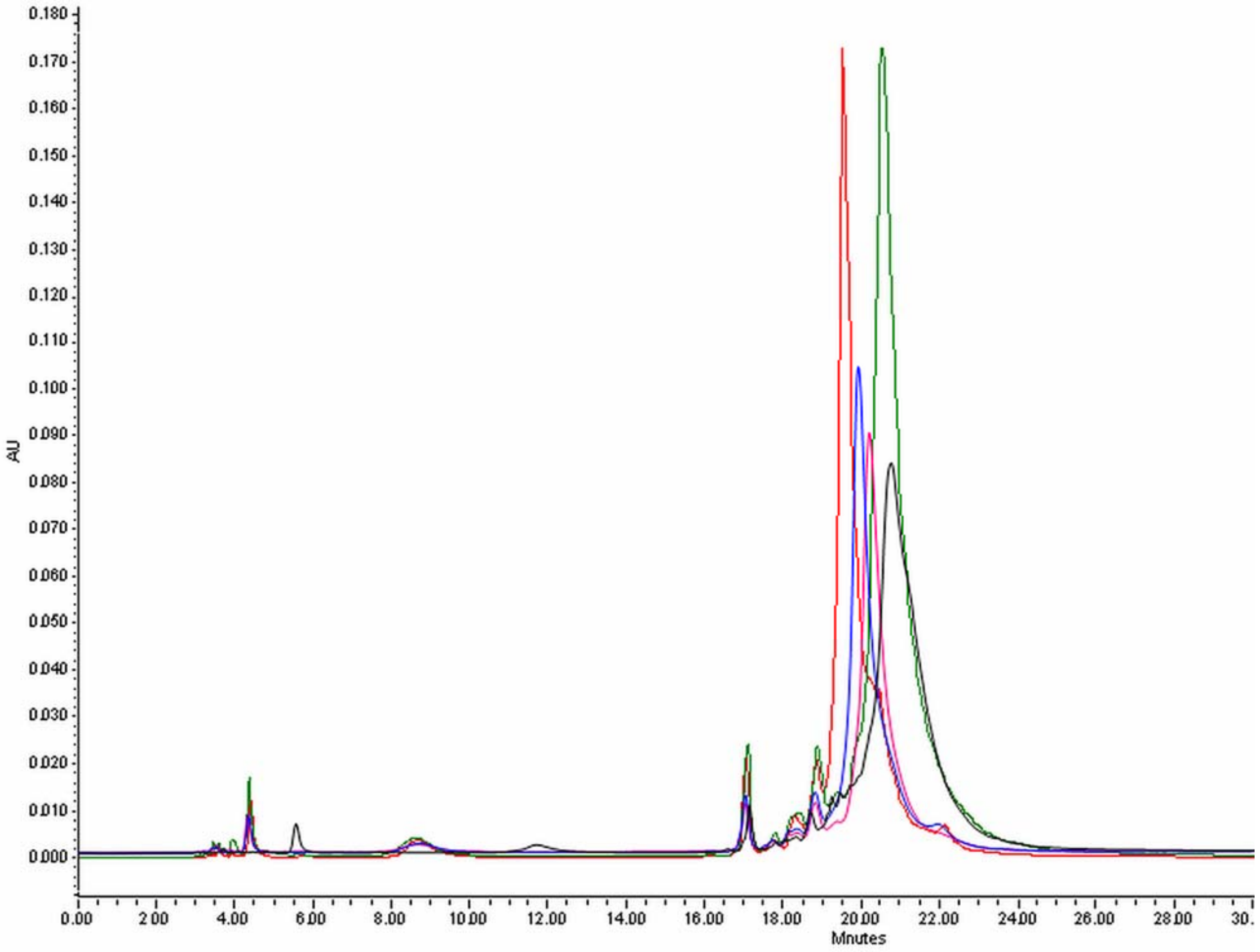

**Figure S-2.** ESI-TOF mass spectrum of the PNA-RPOB15wt 10-mer and its deconvolution. Experimental mass: 2966.16 Da, theoretical mass, 2966.70 Da.

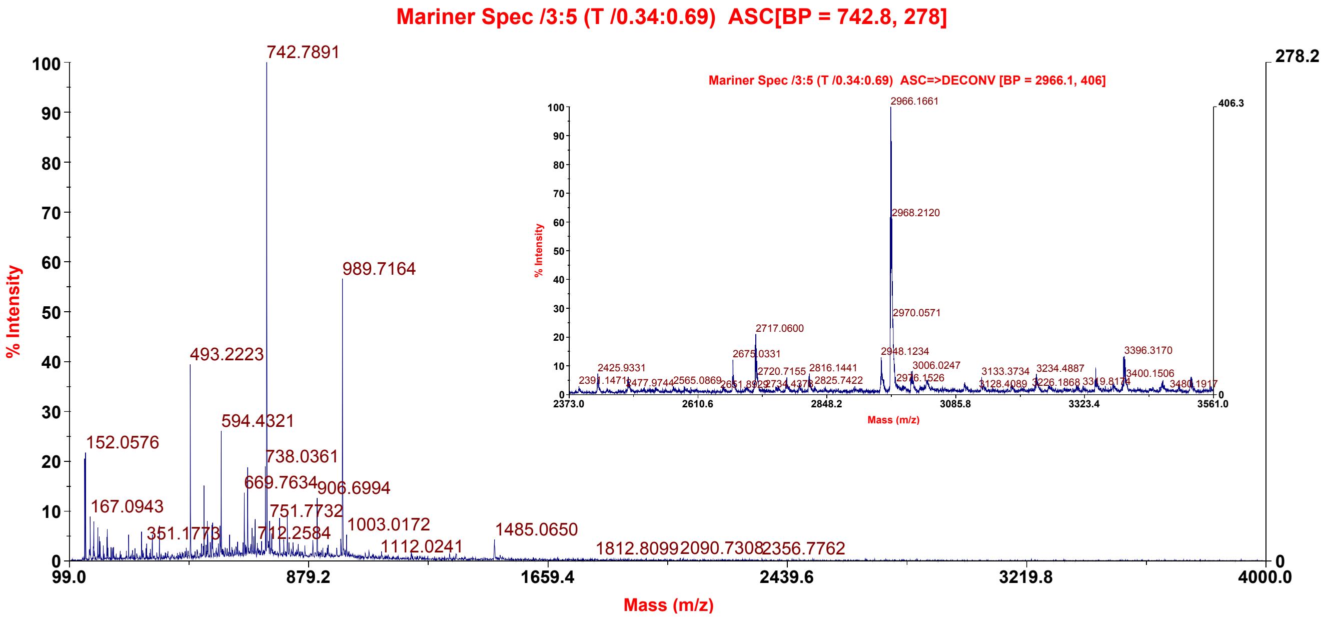


**Figure S-3.** ESI-TOF mass spectrum of the PNA-RPOB15wt 11-mer and its deconvolution. Experimental mass: 3217.42 Da, theoretical mass, 3217.93 Da.

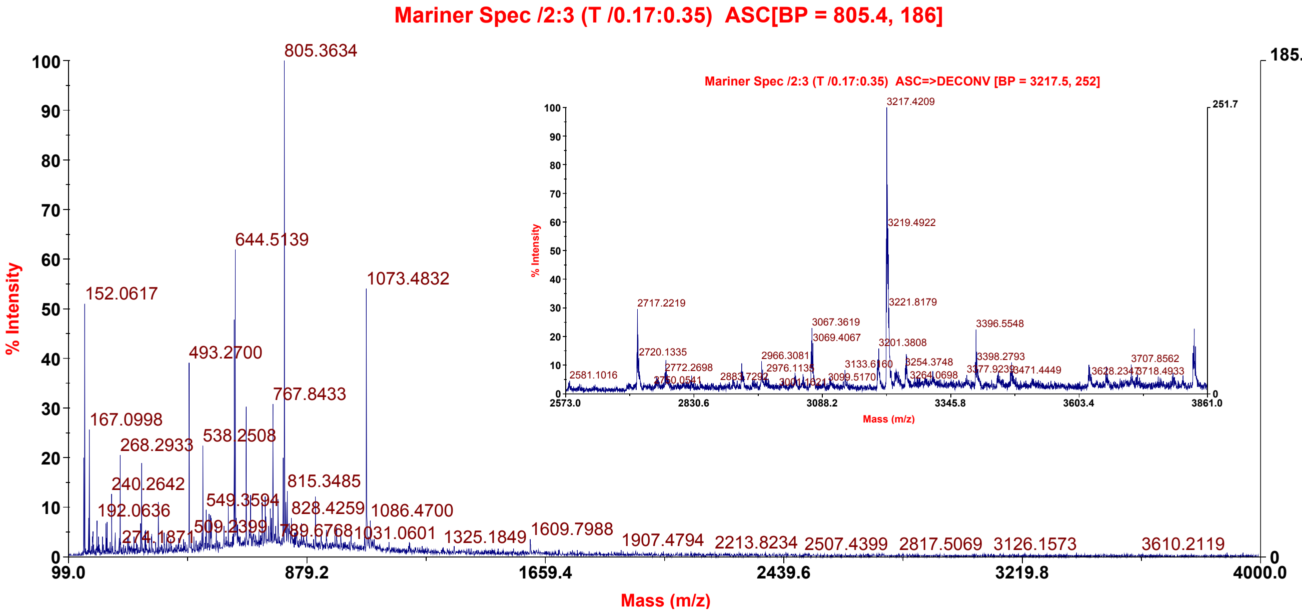

**Figure S-4.** ESI-TOF mass spectrum of the PNA-RPOB15wt 12-mer and its deconvolution. Experimental mass: 3484.36 Da, theoretical mass, 3484.19 Da.

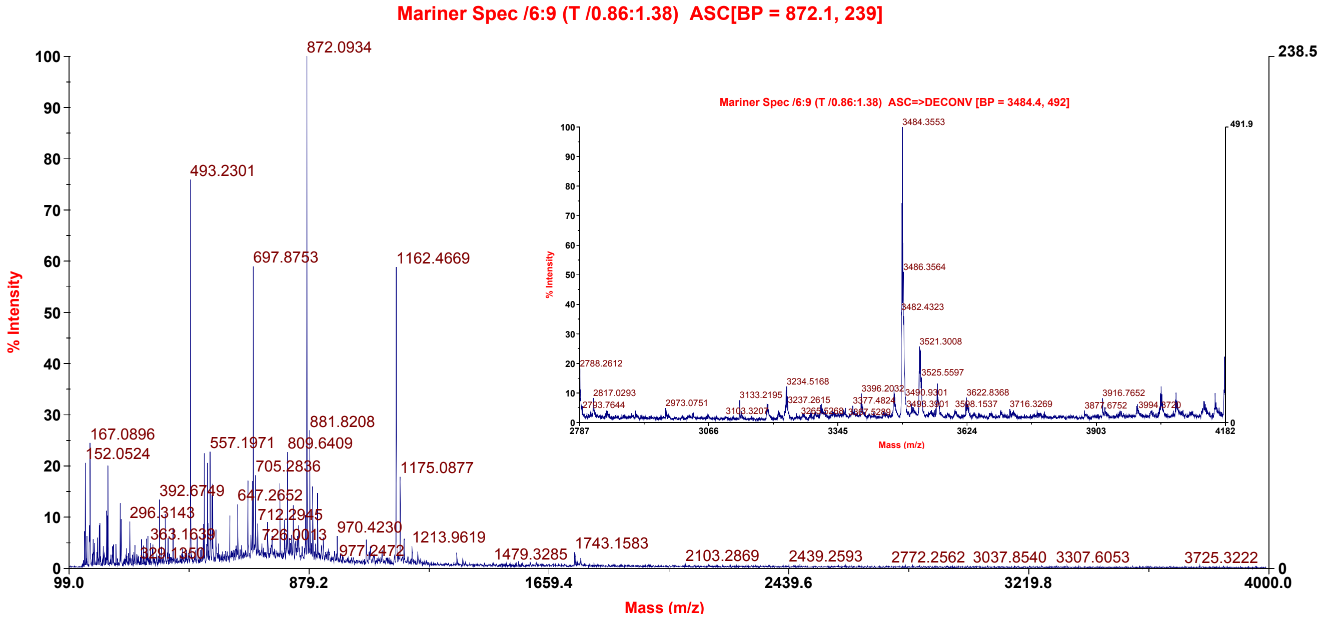


**Figure S-5.** ESI-TOF mass spectrum of the PNA-RPOB15wt 13-mer and its deconvolution. Experimental mass: 3775.42 Da, theoretical mass, 3775.45 Da.

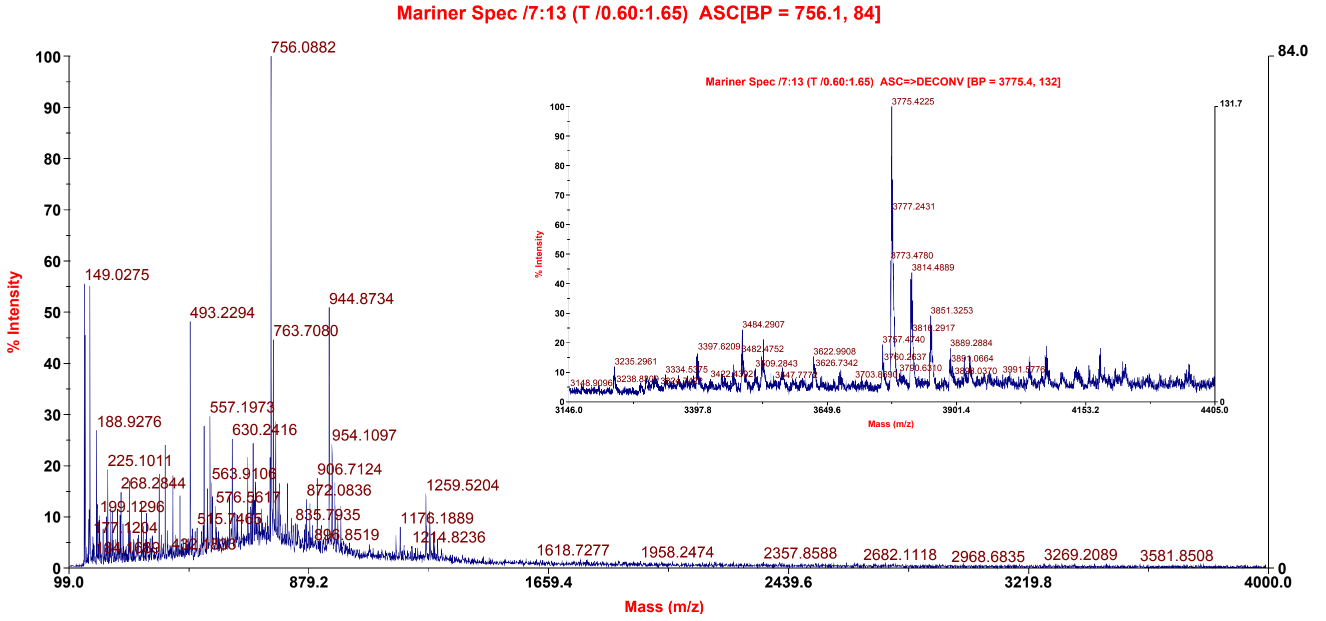

**Figure S-6.** ESI-TOF mass spectrum of the PNA-RPOB15wt 14-mer and its deconvolution. Experimental mass: 4041.70 Da, theoretical mass, 4041.71 Da.

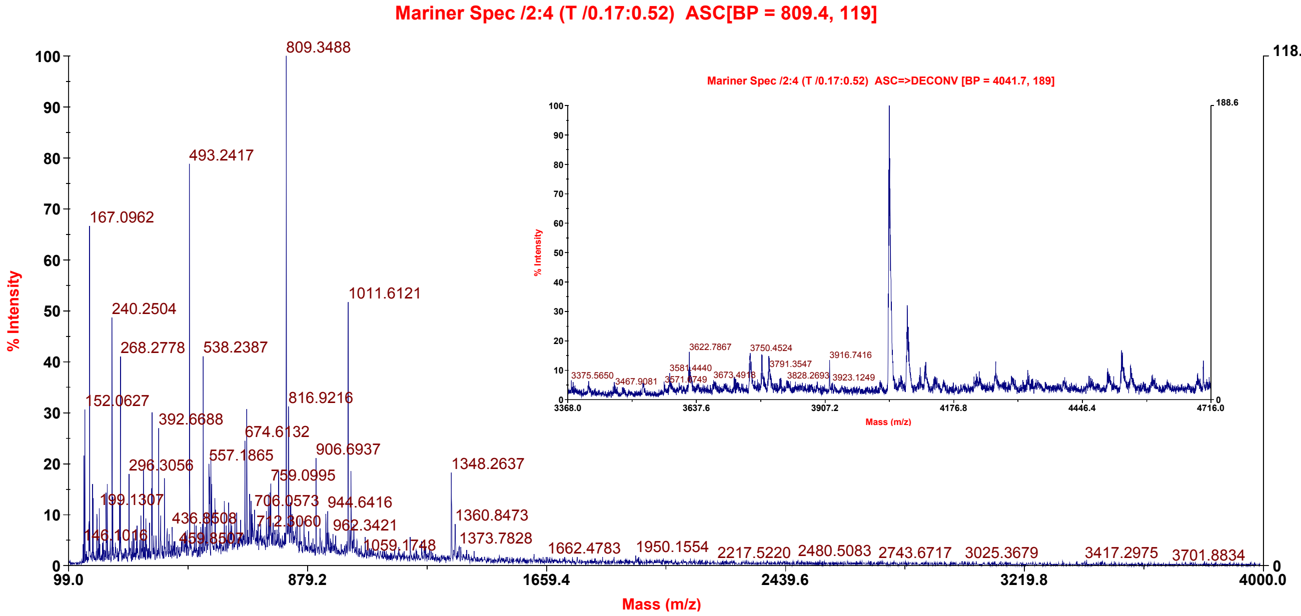


**Figure S-7.** ESI-TOF mass spectrum of the crude PNA-RPOB15wt 15-mer and its deconvolution. Experimental mass: 4292.70 Da, theoretical mass, 4292.94 Da.

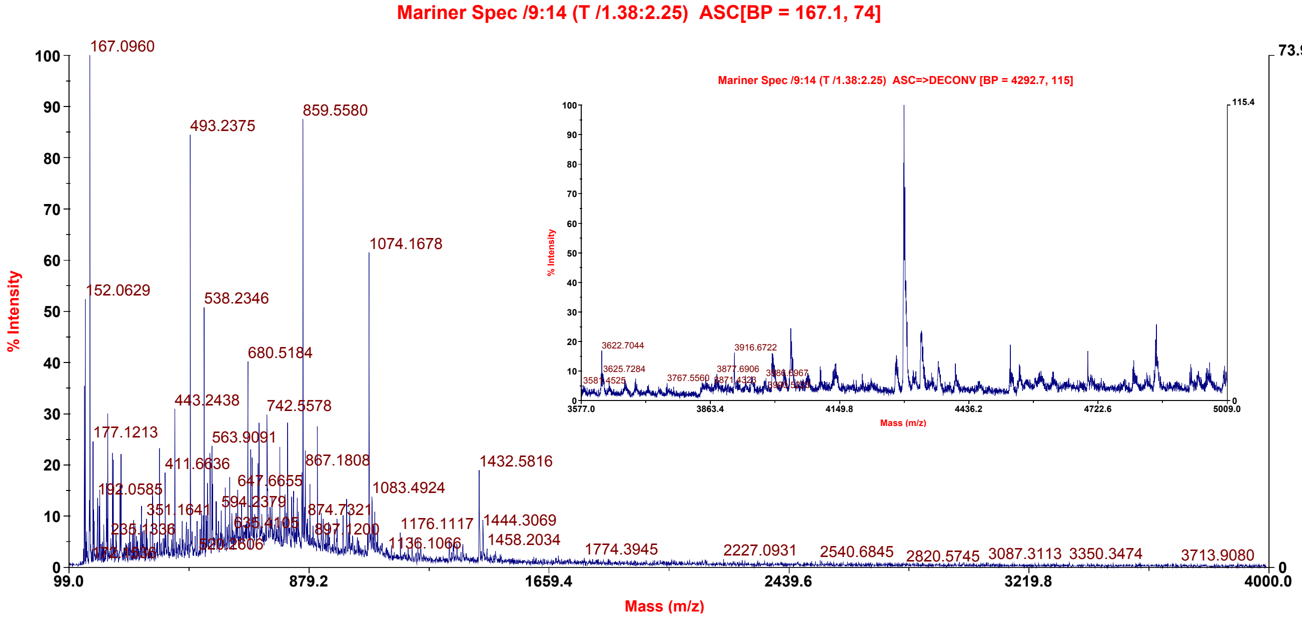

## 2. Solubility of PNA-RPOB15wt in Different Organic Solvents

The solubility of the PNA-RPOB15wt in different organic solvents ($CH_3CN$, MeOH, DMF and DMSO) and aqueous buffers (0.1 M borate, pH 8.0 and 10 mM phosphate, 150 mM NaCl, pH 7.0 (PBS)) was measured (Table S-1) in order to select the most suitable environment for the solution coupling reaction. To this end, small aliquots of the product (about 0.1 mg) were each added of 50 µL of solvent. After 24 h standing at room temperature (under gentle stirring), mixtures were centrifuged at 10.000 g for 5 minutes and PNA concentration in the supernatant was calculated from its content in primary amines, as determined by the fluorescamine assay [Fiche, J.B.; Buhot, A.; Calemczuk, R.; Livache, T. Temperature effects on DNA chip experiments from surface plasmon resonance imaging: Isotherms and melting curves. *Biophys. J.* **2007**, *92*, 935–946]. A calibration curve with glycil-glycine as the reference primary amine was built in parallel and was used for the computation.

**Table S-1.** Solubility of PNA-RPOB15wt (µg/mL) in different environments.

| Solvent or buffer | Solubility of PNA-RPOB15wt (µg/mL) | Solubility of PNA-RPOB15wt (M) |
|---|---|---|
| $CH_3CN$ | 340 | $7.9 \times 10^{-5}$ |
| $CH_3OH$ | 10 | $2.3 \times 10^{-6}$ |
| 0.1 M borate, pH 8.0 | 50 | $1.2 \times 10^{-5}$ |
| DMF | Non soluble | Non soluble |
| DMSO | >3630 | $>8.5 \times 10^{-4}$ |
| PBS | 7 | $1.6 \times 10^{-6}$ |

## 3. Characterization of Fmoc-Cys(Trt)-$PEO_{2KDa}$-COOH and Fmoc-Cys(Trt)-$PEO_{5KDa}$-COOH

The synthesis of two derivatives was performed on a 1 g scale and was carried out following classical procedure of PEO functionalization through amide bond formation in solution. The yield of α-amino conversion was 95% for both PEO derivatives and an overall yield of 60%. The identity of products size was confirmed by MALDI spectrometry (Figure S-8). Gel permeation analysis was also carried out (Figure S-9).

**Figure S-8.** MALDI spectra of Fmoc-Cys(Trt)-PEO$_{2KDa}$-COOH (**A**) and Fmoc-Cys(Trt)-PEO$_{5KDa}$-COOH (**B**).

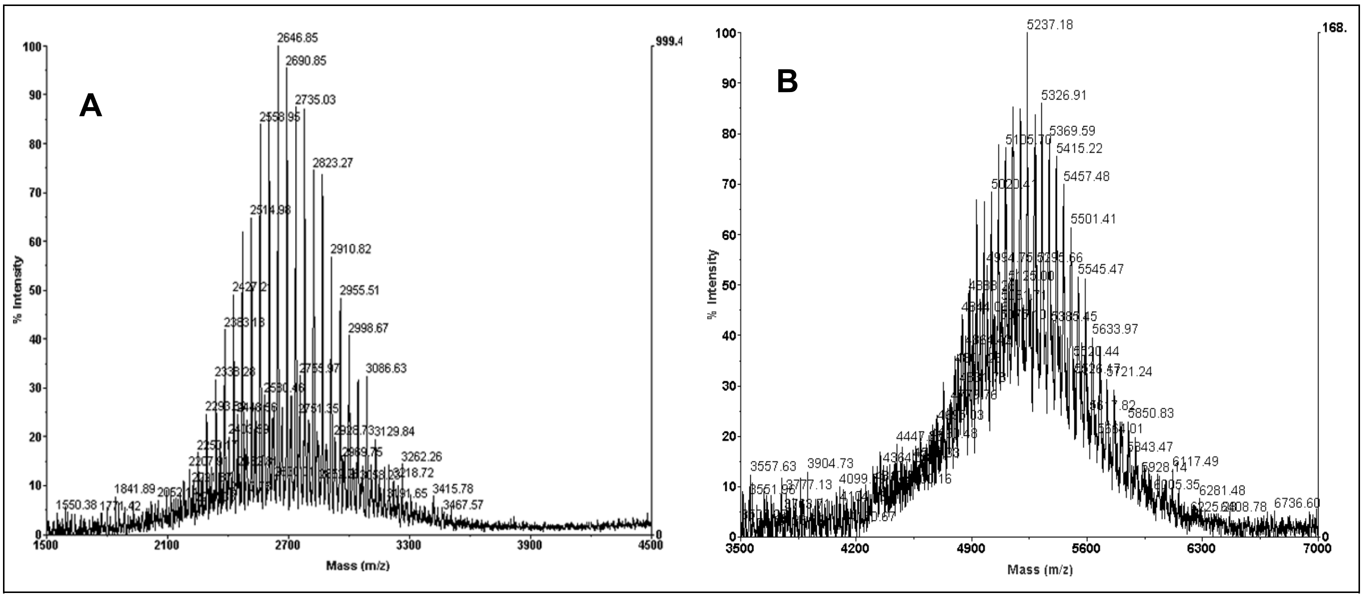


**Figure S-9.** Gel permeation chromatography of Fmoc-Cys(Trt)-PEO$_{2KDa}$-COOH (solid line) and Fmoc-Cys(Trt)-PEO$_{5KDa}$-COOH (dashed line), eluted in Superdex peptide 10/300 GL column; loop 100 µL, T = room temperature, eluent: PBS buffer, flow rate: 0.5 mL/min; detector: readings at 260 nm.

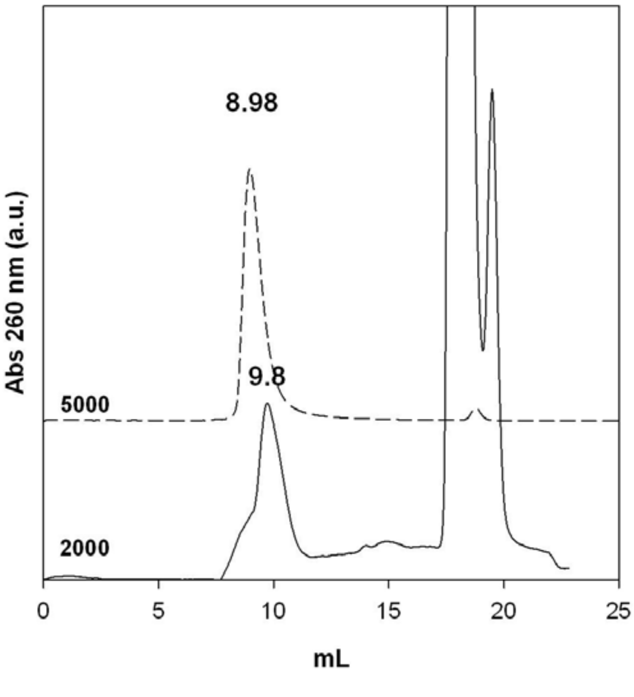

## 4. Conjugation of Fmoc-Cys(Trt)-$PEO_{2KDa}$-COOH to PNA

Conjugation with the 2 KDa PEO derivative was carried out on a smaller scale (1 mg of PNA) using the DMAP/HATU coupling reagents only, obtaining similar results as those with the 5KDa PEO. Figure S-10 shows **residues A** gel permeation chromatography and the MALDI analysis of the NAP5 purified compound.

**Figure S-10.** Characterization of residue A obtained from the coupling reaction with $PEO_{2KDa}$. (**A**) Gel permeation chromatography on Superdex peptide column and (**B**) MALDI analysis of the final product.

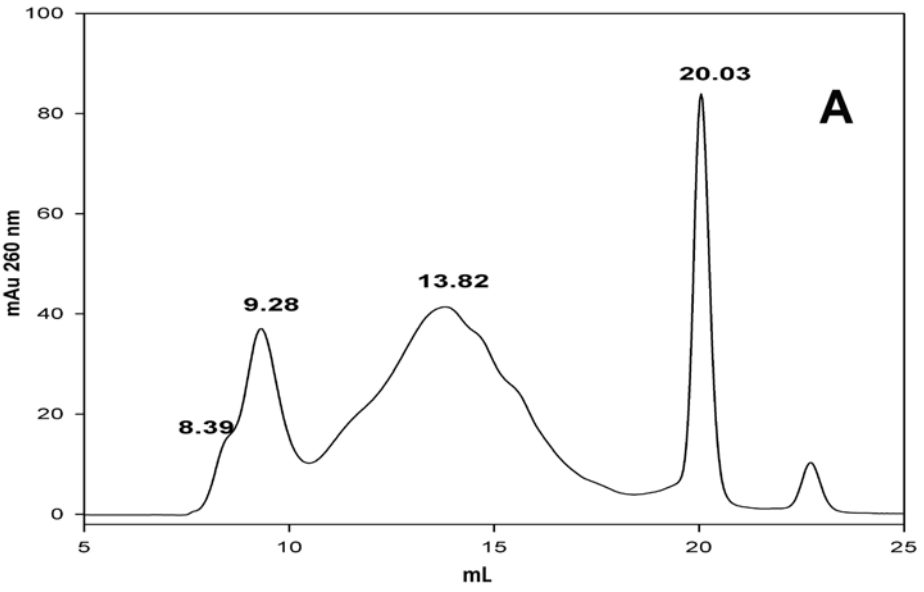


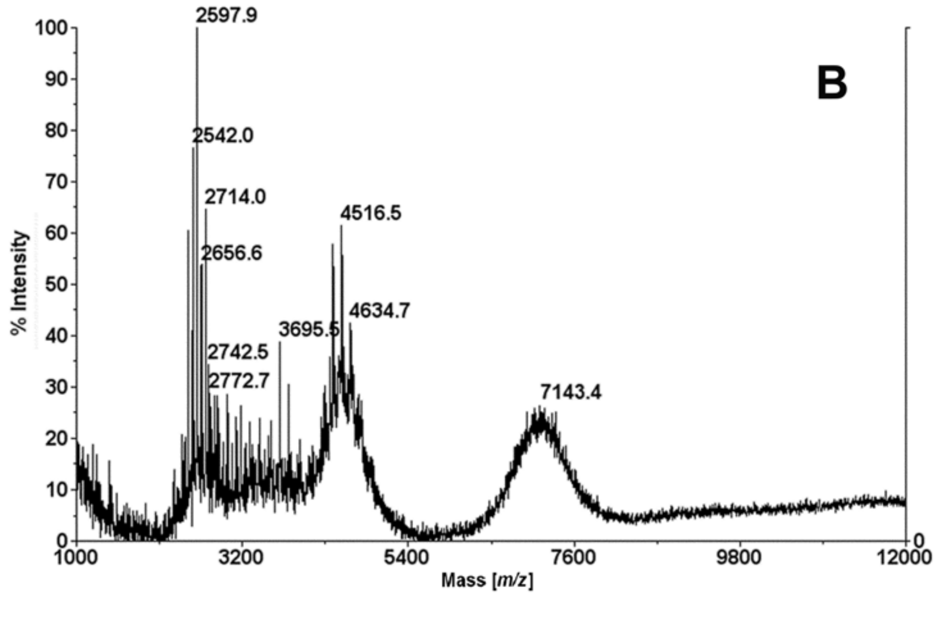

Supplement: Supplementary file 1 [file molecules-17-11026-s001.pdf]
